# Management of intractable epistaxis in patients who received radiation therapy for nasopharyngeal carcinoma

**DOI:** 10.1007/s00405-013-2598-6

**Published:** 2013-07-12

**Authors:** Cheng-Cheng He, Yong-feng Si, Yu-an Xie, Lei Yu

**Affiliations:** 1 The Department of Otolaryngology Head and Neck Oncology, People’s Hospital of Guangxi Zhuangzhu Regional National Autonomy, Nanning, China; 2 Affiliated Tumor Hospitial of Guangxi Medical University, Nanning, China; 3 The Department of Interventional Oncology, People’s Hospital of Guangxi Zhuangzhu Regional National Autonomy, Nanning, China

**Keywords:** Nasopharyngeal carcinoma, Radiotherapy, Intractable epistaxis

## Abstract

To report clinical manifestations, bleeding point localization, and outcomes of management in 16 patients with 16 instances of intractable epistaxis after radiation therapy for nasopharyngeal carcinoma. Retrospective chart review of 16 patients with nasopharyngeal carcinoma (mean age 52.06 ± 14.37 years) with 16 instances of intractable epistaxis during the past 5 years, whose diagnosis was confirmed by angiography (*n* = 10) or MRI/CT imaging studies and clinical manifestations (*n* = 6). The mean radiation dose to the affected carotid artery was 101.37 ± 34.85 Gy. Bleeding points were detected in the internal carotid artery (*n* = 8) or external carotid artery (*n* = 8). Detachable balloons were used in one affected artery for vascular occlusion; six were treated using an absorbable gelatin sponge (*n* = 4) or microcoils (diameter 1 mm) (*n* = 2). Endovascular embolization was successful in seven radiation carotid blowout syndromes with cessation of hemorrhage. One patient underwent external carotid artery ligation and one patient recovered without treatment. The clinical follow-up was 3 months. Therapeutic endovascular embolization of intractable epistaxis is both efficient and safe. It should be considered as the primary treatment modality in intractable epistaxis of nasopharyngeal carcinoma.

## Introduction

Nasopharyngeal carcinoma (NPC) is a common neoplasm in southern China, especially in Guangdong Province and Guangxi Zhuangzhu Regional National Autonomy [1].

The primary treatment for NPC is high-dose radiation therapy (RT), for both the primary tumor and the neck, with promising results; however, potential radiation complications include xerostomia, sinusitis, temporal lobe necrosis, cranial nerve palsy, and brainstem damage, as well as radiation arteritis with carotid stenosis. Intractable epistaxis resulting from radiotherapy alone is a medical emergency. Angiography and endovascular treatment have been used as part of the protocol in management of the condition.

The purpose of this paper is to report the clinical manifestations, bleeding point localization by angiography, endovascular management, and some important related measurements in 16 instances of intractable epistaxis in 16 patients with NPC.

## Methods

We retrospectively reviewed the hospital records of patients with NPC after RT who presented with intractable epistaxis over a 5-year period. In total, 16 patients were recruited for analysis. Patient profiles are listed in Table 1.

**Table 1:** Demographics and outcomes of 16 NPC patients with intractable epistaxis

Number	Sex	Age (years)	Affected artery	Dose (Gy) of affected artery	Number of RT	Angiographic features	Embolic agent	Other therapy	Survival	Follow-up (3 month)
1	F	65	PT ICA	72	1	No	No	TR and NP	DLB	
2	M	54	SS ICA	160	2	PA	No	TR and NP	Alive	5 days
3	M	53	PT ICA	140	2	No	No	TR and NP	DLB	
4	F	43	PT ICA	130	2	No	No	TR	DLB	
5	M	72	PT ICA	72	1	PA	Balloon	TR and NP	Alive	Alive
6	M	61	SS ICA	144	2	PA	No	NP	Alive	2 days
7	M	30	PT ICA	132	2	No	No	No	DLBS	
8	M	58	PT ICA	138	2	No	No	NP	DLBS	
9	M	56	IMA	128	2	MN	No	TR/NP/ECL	Alive	Alive
10	F	45	IMA	74	1	RCB	S	TR and NP	Alive	Alive
11	M	40	IMA	70	1	OR	S	TR and NP	Alive	Alive
12	M	71	IMA	72	1	PA	S	No	Alive	Alive
13	M	35	IMA	74	1	MN	S	NP	Alive	Alive
14	M	71	IMA	70	1	MN	MC	No	Alive	Alive
15	M	52	IMA	72	1	MN	MC	NP	Alive	Alive
16	M	27	APS	74	1	No	No	TR and NP	Alive	Alive

*PT* Petrous, *SS* SpheSinus, *APS* the arteriae pharyngea ascendens of the external carotid artery, *PA* pseudoaneurysm, *OR* osteoradionecrosis, *MN* member necrosis, *ICA* internal carotid artery, *IMA* internal maxillary artery, *TR* tracheostomy, *NP* nasal packing, *ECL* external carotid ligation, *S* sponge, *MC* melt circle, *DLB* died of loss of blood, *DLBS* died of loss of blood and suffocation

Between October 2005 and March 2011, 16 NPC patients were referred to our institute for emergency first aid. We performed a retrospective chart review of these patients. Our study was approved by the Institutional Review Board of the People’s Hospital of Guangxi Zhuangzhu Regional National Autonomy.

All patients in our series had been treated by radiotherapy, three had been treated surgically, four had tumor release, and one had osteoradionecrosis (OR).

The patients were 13 men and 3 women, aged 27 to 72 years, with a mean age of 52.06 ± 14.37 years. The radiation dose to affected arteries varied from 70 to 160 Gy (mean 101.37 ± 34.85 Gy). All patients had oronasal packing/intranasal balloons and blood transfusions to maintain vital signs. In our institute, emergency tracheotomy (usually under local anesthesia) is carried out (*n* = 9) if there is evidence of trismus (signifying difficult intubation) or lack of laryngeal sensation (signifying risk of silent aspiration).

**Fig. 1:**
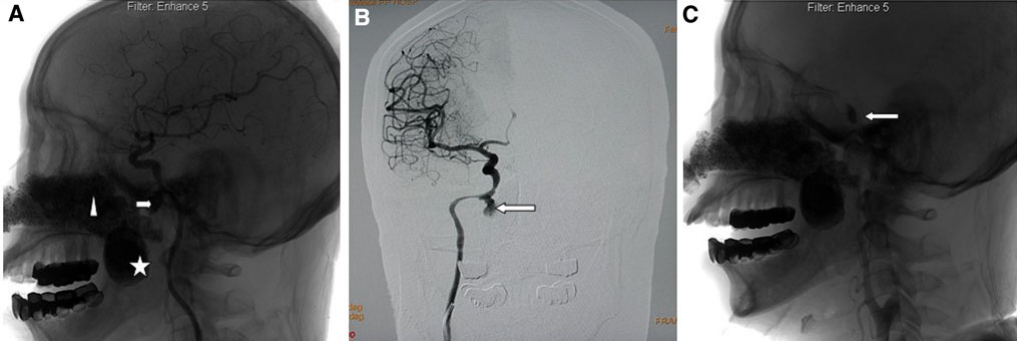
Images of a 72-year-old man (number 5 in the table) who received 72 Gy to treat a primary tumor intractable epistaxis occured 4 months after radiotherapy (RT). **a**, **b** Right lateral carotid angiogram showed radiation arteritis (*arrowheads*) with pseudoaneurysm at the petrous portion of the internal carotid artery (ICA). (Triangle and pentagon show oronasal packing). **c** Postembolization right carotid angiograms revealed total occlusion of the ICA and complete obliteration of the pseudoaneurysm (*arrowhead* shows the balloon)

**Fig. 2:**
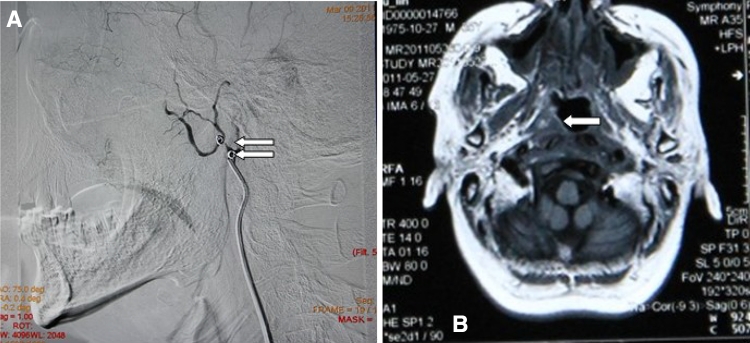
Images of a 52-year-old man (number 15 in the table) who received 72 Gy to treat a primary tumor; intractable epistaxis occured 15 years after radiotherapy. **a** Postembolization internal maxillary artery (IMA) angiograms revealed total occlusion of the IMA (*arrowhead* shows melt circle). **b** *Arrowhead* shows the residual or recurrent tumor

**Fig. 3:**
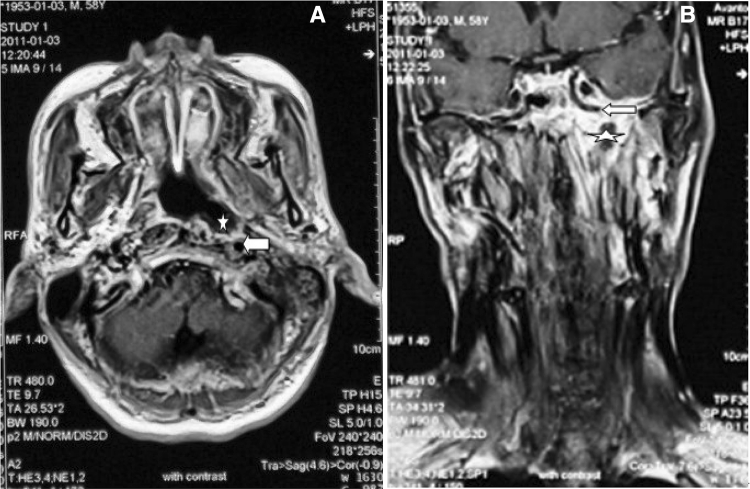
Images of a 30-year-old man (number 7 in the table) who received 132 Gy to treat a primary tumor; intractable epistaxis occured 6 months after radiotherapy (RT). **a**, **b** Horizontal and coronal position of MRI shows the pharyngeal recess (*pentagon*) and petrous portion of the internal carotid artery (ICA) (*arrowhead*)

Catheter angiography through the right femoral approach was carried out in ten cases in which bleeding was stopped by oronasal packing/intranasal balloons. In all ten patients, angiography confirmed epistaxis of the internal carotid artery (ICA) (*n* = 3) (Fig. 1a–c) or internal maxillary artery (*n* = 7) (Fig. 2a, b). The past MRI/CT imaging studies and clinical manifestations confirmed epistaxis of the ICA (*n* = 5) (Fig. 3a, b) in patients who had died of blood loss and choking in a short time (about 10–30 min). The past MRI/CT imaging studies and clinical manifestations confirmed the epistaxis of the arteriae pharyngea ascendens (APS) of the external carotid artery (*n* = 1) [2].

Therapeutic permanent balloon occlusion was performed for vascular occlusion and to obliterate pseudoaneurysm at the ICA (*n* = 1, Fig. 1a–c). Two detachable balloons (Golden Valve, Nycomed, Ingenor, Paris, France) were navigated to the affected arteries and then inflated with contrast media. Before balloon detachment, a 30-min occlusion test was performed (*n* = 1). Six patients with involvement of the internal maxillary arteries (*n* = 6) were treated with absorbable gelatin sponges (*n* = 4) or microcoils (diameter 1 mm) (*n* = 2) for occlusion of the affected arteries and pseudoaneurysms. Postembolization angiograms were routinely obtained to assess the effectiveness of hemorrhage cessation. One patient with involvement of the internal maxillary arteries was treated with external carotid artery ligation (*n* = 1). Hemorrhage ceased in this case.

## Results

Results and follow-up findings are listed in Table 1. Rupture sites included eight ICAs (three cases confirmed by angiography, five confirmed by MRI/CT imaging studies and clinical manifestations). Of eight patients (*n* = 8) who suffered from the bleeding in ICA rupture, seven patients died (*n* = 7) and one patient was alive (*n* = 1). After undergoing detachable balloons used in the affected ICA, the alive patient had transient ischemic attacks, one patients suffered hemiparesis and two people suffered paresthesia, all symptom disappeared in 24 hours but suffered no permanent neurological deficit. Complete cessation of bleeding was achieved in all eight patients who suffered from the bleeding in ECA rupture. Six patients who underwent embolization (*n* = 6), one underwent external carotid artery ligation (*n* = 1), and one patient recovered without treatment (*n* = 1). Of six patients whounderwent embolization, three patients experienced headache (*n* = 3) and two patients experienced low fever (*n* = 2) after the procedure, the above symptoms recovered normal in 24–48 h.

## Discussion

In general, most cases of spontaneous epistaxis occur in the anterior septal area and can be readily controlled by cautery and nasal packing [3]. However, intractable epistaxis occurring in patients following RT for NPC can be difficult to control. Epistaxis can occur because of residual or recurrent disease, neovascularization, or pseudoaneurysm formation, as well as osteonecrosis and soft tissue inflammation. Nasal packing is sometimes ineffective, and surgical ligation of internal maxillary arteries may be unsuccessful because of the presence of collaterals [4]. All patients were managed initially by oronasal packing/intranasal balloons; once immediate bleeding was controlled, endovascular treatment was performed. In other instances, patients died of blood loss and choking. In this investigation, six patients who may have experienced ICA blowout died within about 10 to 30 min. External carotid artery ligation (*n* = 1) was an effective means of stopping internal maxillary artery bleeding.

One ICA blowout was stopped by oronasal packing. We managed this with a detachable balloon that, upon inflation, allowed rapid and complete arterial occlusion and immediate cessation of intractable bleeding. Occlusion was then performed before balloon detachment. For ICA blowout, endovascular treatment would be the only effective means to control the bleeding. Petrous ICA pseudoaneurysms can be treated by main trunk occlusion or a covered stent [5–7]. Concerns about stent erosion and infection because of local inflamed and necrotic tissue, as well as a lack of antiplatelet preparation, would favor main trunk occlusion as the preferred treatment toward, if feasible. The balloon occlusion test with hypotensive challenge is helpful in seeing the adequacy of collaterals. In the series from Low and Goh [8], one main trunk occlusion was carried out for ICA pseudoaneurysm with no further bleeding. In our series, the patient who had undergone balloon occlusion of the ICA did not experience a significant rebleed over 1 year. The method of embolization was not specified. In conclusion, the present series and the contemporary series from Low and Goh [8] suggest that main trunk occlusion after the balloon occlusion test constitutes a safe and effective treatment for patients with ICA pseudoaneurysm.

For internal maxillary artery or external carotid blowout, endovascular treatment is the most effective way to control the bleeding. External carotid ligation can also be an effective and final means to stop bleeding, especially in cases in which the hemorrhage is from the tumor itself and not directly from one of the aforementioned arteries. No patients with massive rebleed died in our series; there was hypervascularity in seven patients, and six patients were treated with selective embolization of the ECA. There was no significant rebleed in the latter six patients. Selective embolization of the ECA, repeated if necessary, was indicated for this group of patients with angiographic hypervascularity.

Using this protocol, we experienced only two rebleeds among the 16 patients managed.

## Conclusion

In irradiated patients with NPC presenting with intractable epistaxis, endovascular main trunk occlusion is the first choice of treatment for those with ICA pseudoaneurysm, and selective ECA embolization is an effective treatment for patients with angiographic hypervascularity.
